# Comparison on Well-Being, Engagement and Perceived School Climate in Secondary School Students with Learning Difficulties and Specific Learning Disorders: An Exploratory Study

**DOI:** 10.3390/bs11070103

**Published:** 2021-07-16

**Authors:** Elisabetta Lombardi, Daniela Traficante, Roberta Bettoni, Ilaria Offredi, Mirta Vernice, Daniela Sarti

**Affiliations:** 1 Department of Psychology, Catholic University of Milan, 20123 Milano, Italy; daniela.traficante@unicatt.it; 2 Department of Psychology, University of Milan-Bicocca, 20126 Milano, Italy; roberta.bettoni@unimib.it; 3 A.R.P. Associazione per la Ricerca in Psicologia Clinica, 20123 Milan, Italy; ilaria.offredi@unimib.it; 4 Dipartimento di Studi Umanistici, Università degli Studi di Urbino “Carlo Bo”, 61029 Urbino, Italy; mirta.vernice@uniurb.it; 5 Fondazione IRCCS Istituto Neurologico Carlo Besta, 20133 Milano, Italy; daniela.sarti@istituto-besta.it

**Keywords:** specific learning disorder, learning difficulties, well-being, school engagement, school climate

## Abstract

Reading and writing skills influence the social status of students, exerting effects not only on learning, but also on wellbeing. This study aimed to assess the impact of diagnosis of specific learning disorder on well-being in secondary-school students, comparing students with a diagnosis of specific learning disorder (SLD-group), students showing learning difficulties without diagnosis (LD-group) and students without learning difficulties (control-group). Students were tested with neuropsychological screening tests in order to identify learning difficulties and were further assessed by means of psychological and school well-being questionnaires. The results show that LD group perceive themselves as having a low sense of mastery and autonomy, less interest and engagement in daily activities and low peer social support than their schoolmates. This result highlights, for the LD group, a low well-being experience, which is not observed in the SLD and control groups. On the contrary, SLD group students do not differ from control group students in any dimensions except for the perceived parents’ support and involvement in school life, in which the SLD group show the highest scores. This work underlines the importance of having a diagnosis as it seems to work as a protective factor for both the psychological and school well-being of the student.

## 1. Introduction

In recent decades, there has been a growing interest in students’ well-being at policy level, and this attention led research to focus on the study of the psychological aspects related to learning processes. In this light, several studies aimed at discovering whether and how education systems might support student’s well-being [1,2]. The Program for International Student Assessment (PISA) defines well-being as the quality of students’ life within various dimensions of well-being, including life as a whole, self-related, school-related, and out of school well-being [3]. Interest in high school students and particularly 15-year-olds is significant to the PISA and it is meaningful to address how this population feels about their experience of well-being at school. Seligman (2011) outlines a new dynamic concept of well-being, according to which the complex nature of human flourishing is composed of several concepts and experiences correlated with each other: positive emotion, engagement, relationships, meaning, and accomplishment (PERMA). In positive psychology applied to educational contexts, several studies focused, in particular, on engagement in school activities, which has been described as a multidimensional construct [4,5]. It involves affective or emotional components, as it refers to the young people’s interest and joy regarding school with the presence of positive emotions [6]. Moreover, it involves behavioural components, which refer to attention, effort, and persistence in accordance with school expectations [6]. Finally, it involves cognitive aspects, referring to the strategies applied to learning activity and to self-regulated learning [4,7].

There is evidence of a relationship between support from parents, peers and teachers and affective school engagement [8,9], as the support from others was found as a key predictor of school engagement. Furthermore, there is evidence that the student’s engagement with school is significantly influenced by the school climate perceptions [10,11]. School climate reflects norms, goals, values, interpersonal relationships, teaching and learning practices, and organizational structures [12]; a positive school climate supports the student’s engagement in school activities [13,14]. School climate perception is important for students’ positive experience of life and exerts a significant influence on their engagement with school [10,11], but although school climate has been considered an important factor in supporting engagement in school activities, its influence is effective with the moderation effect of the students’ experience of well-being [15].

School is one of the most important developmental contexts in young people’s lives and can be a key source for the acquisition of the skills and competencies that support a student’s ability for a successful adaptation [16,17]. Moreover, schools provide accessible and relatively stable sites to undertake interventions to promote well-being [18]. Hence, schools are uniquely placed to promote the well-being not only of young people, but also of the school communities, from a broader point of view [19]. As schools are central to students’ physical and mental health, a whole-school commitment to create a nourishing environment and to cultivate well-being is imperative.

A focus on flourishing in schools is particularly important because childhood and adolescence are pivotal stages of development, which carry implications for functioning over the life-course. Adolescence is often viewed as a critical stage in the emergence and trajectory of mental illness [20], and rates of mental health problems, especially depression and anxiety, are consistently reported as problematically high in this developmental phase [21].

In summary, the relationship between school climate, student engagement, and well-being is well defined in the literature [15]. Moreover, there is evidence that these dimensions appear to be related to each other and to literacy skills [14,22], and a positive correlation between high-school students’ well-being and their academic achievement was observed [22,23]. A recent study [24] showed that students’ well-being (in particular, the perception of support from the others and the possibility to ask help when needed) is fundamental to promote the experience of social inclusive schools’ environments, especially for students with specific learning disorders.

Specific learning disorders (SLD) (DSM-5; American Psychiatric Association, Washington, DC, USA, 2013) are neurodevelopmental disorders characterized by difficulties in specific academic areas such as reading, writing, mathematics, and spelling. SLD are diagnosed in individuals with normal intelligence, no neurological and sensory deficits and with adequate educational and socio-cultural opportunities [25]. In Italian schools, 3.1% of students have been diagnosed with SLD, such as dyslexia, dyscalculia, and handwriting and spelling disorders, as shown by the last report edited by the Italian Ministry of Education [26]. The data for secondary school show 5.3% of students with SLD compared to the total number of students attending these schools [26].

Several empirical studies have suggested that SLD is frequently associated with social-emotional and behavioral problems and can cause intense emotional suffering [27,28], such as an increased risk of developing externalizing and internalizing problems, loneliness, and poor self−esteem [29,30,31], with effects on well-being.

The study by Walker and Nabuzoka (2007) compared the academic achievement and social functioning of children with and without SLD and found that academic achievement is related to social functioning. Other studies [32,33] have shown that early reading and writing difficulties are a risk factor for the development of both internalizing problems in the early years of schooling and for externalizing problems in the following years, in particular in adolescence [34]. Generally, it is assumed that learning difficulties have profound effects on a child’s emotional life. Students who have difficulties in developing literacy often experience feelings of deep distrust in their own abilities, low motivation, and low self-esteem. Additionally, they are afraid to participate in activities, because they anticipate their own failure. Accordingly, reading and writing skills have an impact on social status recognition within the classroom group [35,36,37], with significant effects not only on learning, but also on well-being and school engagement.

A recent study [38], comparing students with SLD and typically developing children, found closer relationships between the learning and emotional problems at school and internalising/externalising symptoms in the SLD primary school students; moreover, this highlights the importance of a supportive school and family environment to improve the school well-being and general psychological health of these children. In this vein, a study conducted with adolescents with dyslexia highlighted that they experience low academic and social self-efficacy, low mood, and a loss of hope and motivation when they are faced with higher scholastic tasks [39]. These results suggest that these problematic dimensions, strictly connected with the student’s well-being, have a cumulative effect on development, thus demonstrating that they persist when they grow. All these dimensions appear more relevant in the adolescence period of life, in which the recognition of the risk factors for the well-being of students is crucial. In fact, when learning difficulties meet the typical problems of adolescents, further complications could arise.

In Italy, in 2010, a law was promulgated (L. 170/2010) to protect the educational rights of students with SLD, according to the inclusive perspective promoted by the *International Classification of Functioning, Disability and Health-Children and Youth*, (ICF-CY, [40]). In this framework, the social and physical environment plays a major role in promoting the best functioning of a person. When people live/work/study in an environment fit for their abilities and attitudes, they can increase self-efficacy and self-esteem and, in general, well-being. Italian students with a diagnosis of SLD can use compensatory strategies and devices (word processors, calculators, digital dictionaries, conceptual maps) and are given more time to complete tests. All of these support mechanisms allow them to adequately cope with the demands of school and, in this way, students are able to obtain appropriate levels of learning, through the recognition and the opportunity to address these difficulties that mainly happen when they are under pressure [41]. In addition, De Boer et al. (2011) found that teachers do not feel confident in their ability to teach students with learning disorders. Therefore, these students encounter teachers who do not feel effective in supporting the abilities of students with learning disorders and may feel that any type of teaching practice can be effective. Thus, in this vein, they often do not use alternative instructional practices related to, for example, creativity or dynamism, strong points in the cognitive functioning of children with SLD [42]. For these students, the choice of attending secondary school is correlated with the severity of the disorder (difficulty in reading, spelling, and mathematics), IQ, and access to extracurricular activities and appropriate study guidance [43,44]. Most of these students attend vocational schools because high schools (in humanities or scientific topics) are too challenging for them.

It is worth noting that, beyond the students with a certified SLD, there are many students who show learning difficulties, but have never undertaken a diagnosis procedure; indeed, the incidence of SLD is widely underestimated [45]. In particular, learning disorders are poorly recognized in secondary-school students, because they have already compensated for—or are trying to compensate for—their difficulties at this stage of schooling [46]. For that reason, these students with difficulty in learning but without any diagnosis cannot gain advantage from the Italian law targeting students with SLD nor from the social recognition of their difficulties, especially by teachers. They constantly realize that their own school performance does not conform to that of their peers and usually live under pressure throughout the school-learning experience [41]. This condition is associated with negative feelings, such as low self-esteem and anger, disengagement from learning activities and school drop-out in their later school career. Indeed, learning difficulties often result in dropouts during high school and a related loss of fulfillment of one’s social and work opportunities [47]. This is more significant when considering those students who are not recognized within the school environment as having a special need, but are merely stigmatized as not being at all interested in education.

No prior study, to the best of our knowledge, has focused on the well-being experience of these struggling students. The present study aimed to assess the impact of diagnosis of SLD on well-being experience in secondary-school students, comparing students with a diagnosis of SLD, students showing learning difficulties without diagnosis and students without learning difficulties.

More specifically, this work pursues two aims:

1. to assess the relationship between student engagement, well-being and perceived school climate in secondary school students with different literacy skills;
2. to assess the role of literacy skills and of diagnosis of SLD on well-being, perceived school climate and engagement in high-school students.

## 2. Materials and Methods

### 2.1. Participants

The present study originates from a larger study in which a total of 157 students (*M_age_* = 15; 6 years, *SD* = 6.2 months; Female = 57%) attending the 10th grade of secondary schools in the northern part of Italy were tested. Twenty-one students out of the total participants (13%) were certified with a diagnosis of a specific learning disorder (SLD). Such a high rate of students with a SLD could be due to the fact that the recruitment took place in vocational schools, where there usually is a percentage of students with specific learning disorders higher than the national rate (5.3%). Moreover, testing literacy skills in vocational secondary schools, which, in general, are attended by students who are less proficient in such abilities than their peers attending high schools specialized in humanities or scientifics, led to the identification of a group of students with learning difficulties, but without any SLD diagnosis. Therefore, data from the assessment procedure, carried out on the whole sample (*N* = 157), allowed us to identify three experimental groups matched by gender, age and non-verbal intelligence, but with different literacy skills: (i) a group of students with learning difficulties (LD: *N* = 14, 8 girls; *M_age_* = 15; 8 years, *SD* = 6.8 months) who have not gone through any diagnosis procedure and without a clinical diagnosis of a specific learning disorder; (ii) a group of students with a diagnosis of a specific learning disorder (SLD: *N* = 14, 9 girls; *M_age_* = 15; 7 years, *SD* = 6.4 months); (iii) a control group without learning difficulties (CG: *N* = 14, 10 girls; *M_age_* = 15; 5, *SD* = 3.5 months).

The inclusion criteria for LD group were: literacy scores < 1.5 *SD* from the normative mean in at least 2 tests or scores of 2 *SD* below the normative mean in at least 1 test of literacy skills assessment. Participants in the SLD group were selected from the group of 21 students with a diagnosis, in order to have a group matched to LD group for gender, age, and nonverbal intelligence. SLD group showed the following specific learning disabilities, according to ICD-10 ([48] the *International Classification of Diseases*, 10th revision): specific reading disorder (ICD10 code: F81.0, *N* = 5), mixed disorder of scholastic skills (ICD10 code: F81.3, *N* = 4), specific disorder of arithmetical skills (ICD10 code: F81.2, *N* = 3), specific spelling disorder (ICD10 code: F81.1, *N* = 1) and disorder of written expression (ICD10 code: F81.81, *N* = 1). In the control group, literacy skills were within the normal range.

Participants came from the middle class, according to the *Family Affluence Scale*–FAS [49]. Furthermore, all students were monolingual Italian native speakers.

### 2.2. Measures

#### 2.2.1. Non-Verbal Intelligence

Non-verbal intelligence was assessed by *Standard Progressive Matrices*-SPM, [50].

#### 2.2.2. Literacy Assessment

Decoding ability was assessed by speed and accuracy in reading a list of pseudo-words (DDE-2 [51]); reading speed in pseudo-words was measured by the number of syllables per second; reading accuracy was measured as the number of errors in reading aloud.

Reading comprehension was evaluated through the Advanced MT 2, a standardized text reading test [52]. Students were presented with 10 multiple-choice questions, after reading the text silently. The score was the number of correct answers.

Accuracy in spelling was assessed through the Advanced MT 3, a text dictation test [53]. The test required dictation at a constant rhythm of one word every 2 s. The score was the number of incorrectly written words.

A series of preliminary analyses showed that the LD and SLD groups did not significantly differ for the literacy assessment variables, as shown in Table 1, describing means and standard deviations for scores at the Raven’s Progressive Matrices and z-scores on the literacy skill tests for each of the three groups.

#### 2.2.3. Well-Being

The *Comprehensive Inventory of Thriving-CIT* [54,55] is a questionnaire assessing the general well-being experience through 54 items, pertaining to seven principal dimensions. The reliability indexes for all the 157 participants are reported in brackets for all dimensions. The seven dimensions are: relationships (composed of support, community, respect, loneliness, belonging and trust scales, α = 0.83) (e.g., “There are people who give me support and encouragement”), engagement (e.g., “I get fully absorbed in activities I do”, α = 0.60), mastery (composed of skills, learning, accomplishment, self-efficacy and self-worth scales, α = 0.87) (e.g., “The work I do is important for other people”), autonomy (e.g., “Other people decide most of my life decisions”, α = 0.61), meaning (e.g., “I know what gives meaning to my life”, α = 0.70), optimism (e.g., “I expect more good things in my life than bad”, α = 0.64), subjective well-being (composed of life satisfaction, positive feelings, and negative feelings, α = 0.88) (e.g., “I am satisfied with my life”). The reliability of the total scale is α = 0.93). We reversed the items negatively phrased, pertaining to the scales autonomy, loneliness and negative feelings. The rest of the items were phrased in a positive direction such that high scores mean that respondents view themselves positively in important areas of functioning. Students were instructed to respond on a 5-point Likert scale to each item. Mean scores for each subscale and for the total score were carried out (range: 1–5).

#### 2.2.4. Engagement

The Italian adaptation of the *Student Engagement Scale–SE* [5,56] is a questionnaire that assesses three dimensions of student engagement: affective, behavioral and cognitive engagement. The reliability indexes for all 157 participants are reported in brackets. The affective engagement scale measures students’ positive inclination and interests for learning and school (e.g., “I think what we are learning in school is interesting”, α = 0.86); the behavioral engagement scale explores students’ involvement both in school and extra-curricular activities and the effort in learning (e.g., “In class I work as hard as I can”, α = 0.87). The cognitive engagement scale assesses students’ investment in learning processes and strategies (e.g., “Make up my own examples to help me understand the important concepts I learn from school”, α = 0.93). Students were asked to indicate their level of agreement on a 7-point Likert scale. The mean score for each subscale was carried out (range: 1–7).

#### 2.2.5. School Climate

School climate was evaluated by the *Georgia School Climate Survey* (GSCS). The survey was developed by the Georgia Department of Education (GADOE) Assessment and Accountability Division, the Georgia Department of Public Health, and Georgia State University. The questionnaire is composed of 20 items related to the following areas (the reliability indexes for all the 157 participants are reported in the brackets): school connectedness (α = 0.57), peer social support (α = 0.64), adult social support (α = 0.70), cultural acceptance (α = 0.71), social/civic learning (α = 0.53), physical environment (α = 0.67), school safety (α = 0.51), peer victimization (α = 0.72), order and discipline (α = 0.61), and parents’ involvement (α = 0.75). The reliability of the total scale (α = 0.80) is in line with the recent literature [57]. The items of the questionnaire were administered after being translated in Italian and back-translated by an English native speaker. Answers were given on a 4-point Likert scale. Mean scores for each subscale were carried out (range: 1–4).

### 2.3. Procedure

Caregivers and the students were informed of the aim and procedure of the study, after receiving the school-manager’s approval to carry out the research project. Both parents provided written consent for their child’s participation in the study and students gave informed written consent to the study, according to the General Data Protection Regulation (GDPR 2016/79, 25/05/2018). The questionnaires, the SPM, the text reading test and the dictation were administered in the classroom, in two sessions, whereas the decoding abilities were assessed in one individual session in a quiet room at school. The present study was approved by the Scientific and Ethics Committee of the Department of Psychology of Catholic University of Milan, in accordance with the Helsinki Declaration.

### 2.4. Data Analysis Strategy

The small sample size of each group suggested the use of non-parametric statistics. The Spearman’s rho coefficients were computed to determine the correlations between engagement dimensions, school climate, and well-being for each of the three groups of participants. Kruskal–Wallis’ ANOVAs were carried out to assess the differences within the specific learning disorder group (SLD), the learning difficulties group (LD) and the control group (CG) regarding well-being experience, perceived school climate and student engagement. Mann–Whitney tests were applied to investigate post hoc comparisons, and effect size measures were carried out to add information on the reliability of the observed differences.

## 3. Results

Spearman’s rank-order correlation analysis showed different patterns of results for each group (Table 2): for the control group, there is no significant association between well-being, school engagement, and school climate perception; as for SLD students, well-being is positively related to the affective and behavioral scales of engagement, whereas for LD students, well-being is positively related to all three dimensions of the student engagement construct and to school climate. The latter variable is also related to the behavioral and affective engagement.

Comparisons between groups in engagement, well-being and perceived school climate scores, carried out through Kruskal–Wallis’ ANOVAs, showed the following results.

As for well-being experience (CIT), data showed that there were significant differences between groups in the engagement [*χ^2^*(2) = 6.56, *p* = 0.02, *η^2^* = 0.16], mastery [*χ^2^*(2) = 4.94, *p* = 0.04, *η^2^* = 0.12] and autonomy [*χ^2^*(2) = 4.65, *p* = 0.05, *η^2^* = 0.11] scales. As shown in Table 3, the scores were significantly higher for the SLD group than for the LD group on all the three scales: engagement [*U* = 41.5, *p* = 0.004, *ES* = 1.01, 95% *CI* (1.85, 0.254)], mastery [*U* = 49.5, *p* = 0.013, *ES* = 0.84, 95% *CI* (1.66, 0.107)], and autonomy [*U* = 52.5, *p* = 0.017, *ES* = 0.81, 95% *CI* (1.591, 0.052)]. The scores were significantly higher for the control group than for the LD group only on the engagement scale [*U* = 62, *p* = 0.047, *ES* = 0.68, 95% *CI* (1.387, 0.12)].

Data from the *Student Engagement* questionnaire (SE) showed a significant difference between groups only on the behavioral engagement scale [*χ^2^*(2) = 6.22, *p* = 0.02, *η^2^* = 0.15), as the scores were significantly higher for the control group than for the LD group [*U* = 30.5, *p* = 0.005, *ES* = 0.98, 95% *CI* (2.190, 0.494)].

Differences between the SLD and the control group were not significant regarding either the well-being or the student engagement dimensions.

The perceived school climate questionnaire (GSCS) showed significant differences in the school connectedness [*χ^2^*(2) = 6.51, *p* = 0.02, *η^2^* = 0.16], peer social support [*χ^2^*(2) = 5.24, *p* = 0.03, *η^2^* = 0.13] and parents’ involvement [*χ^2^*(2) = 9.24, *p* = 0.005, *η^2^* = 0.23] scales. Scores were significantly higher for the SLD group than for the LD group on peer social support [*U* = 65.5, *p* = 0.05, *ES* = 0.86, 95% *CI* (1.317, 0.180)] and parents’ involvement (*U* = 57, *p* = 0.023, *ES* = 0.78, 95% *CI* (1.492, 0.028)], whereas the scores were significantly higher for the control group than for LD group on peer social support [*U* = 50, *p* = 0.011, *ES* = 0.93, 95% *CI* (1.650, 0.098)], and school connectedness [*U* = 45.5, *p* = 0.006, *ES* = 1.035, 95% *CI* (1.758, 0.183)] scales. The LD group showed scores higher than the control group on adult support [*U* = 59.5, *p* = 0.035, *ES* = 0.73, 95% *CI* (1.439, 0.074)]. The only significant difference between the SLD and control group concerns the perceived parental involvement, where SLD students showed scores significantly higher than the control group [*U* = 37, *p* = 0.001, *ES* = 0.84, 95% *CI* (1.97, 0.45)].

## 4. Discussion

The aim of the present study was to assess the role of learning difficulties/disorders on well-being experience, engagement in learning activities, and perceived school climate in secondary school students, taking into consideration also the presence of a diagnosis of SLD, which in Italy can offer protection of the student’s educational rights. More specifically, the novelty of this preliminary study is to focus on a particular population, i.e., students with learning difficulties, but without a diagnosis of specific learning disabilities.

The correlational analysis showed that, for students with learning difficulties (LD), well-being experience is positively associated with all dimensions of student engagement, i.e., a positive inclination and interest in learning and school (affective engagement), involvement in the learning processes (behavioral engagement), and investment in learning processes and strategies (cognitive engagement). Furthermore, a positive school climate perception is related to both behavioral and affective engagement. These results for the LD group show that school context, student engagement, and well-being are interrelated. In students with certified specific learning disorders (SLD) the positive well-being experience is directly associated only with the positive emotions that students experience with respect to school activities (affective engagement) and with students’ effort in learning (behavioral engagement). On the contrary, in typically developing adolescents, no association was found between well-being, student engagement and school climate, demonstrating the independence of these dimensions in the absence of learning difficulties or disorders.

The comparisons within the three groups regarding each dimension of well-being, engagement and school climate revealed that students with learning difficulties, but without any diagnosis (LD), perceive themselves as having a low sense of mastery and a low sense of autonomy, compared to their schoolmate with certified specific learning disorders. They also experience significantly less interest and engagement in daily activities than their schoolmates (both SLD and control groups). The school climate is perceived by the students with learning difficulties as being poor due to low peer social support. This result highlights, therefore, for the LD group, the perception of a climate of loneliness, which is not observed in the SLD and control groups. However, the LD group reports a higher level of adult social support than the control group. This evidence highlights that LD students, despite of their being more isolated than their peers, trust the reference adults who are in their school community. They seem to be more sensitive to the fairness and supportiveness of the adults than the students in the control group, who are likely not to note such support, as they already feel the context comfortable for them, especially because of their good relationships with the peers.

On the contrary, students with specific learning disorders do not differ from the control group in any dimensions except one, the perceived parents’ support and involvement in school life, in which the SLD group show the highest scores. This finding highlights that students with specific learning disorders are more likely to ask for help from parents and teachers and they feel the need to be adequately supported and accepted by them [58]. The pattern of results observed in the SLD group is in line with the literature showing that the effects of parental involvement are manifold: children whose parents are more involved in school activities show fewer social problems and better social skills, better school outcomes, and lower dropout rate [59,60,61]. This is particularly significant if we consider that the most salient predictors of school dropout among secondary students with learning difficulties/disorders includes parent’s expectations and the quality of students’ relationship at school [62]. Some authors demonstrated that parental involvement is the key to the acquisition of better school performance and academic outcomes [63,64,65,66]. In particular, the tendencies to ask for help when needed and to trust others seem to mainly be important for adolescents with specific learning disorders.

In conclusion, this work underlines that when learning difficulties are present, notwithstanding a good cognitive level, in the Italian context, where there is a law aimed at promoting inclusive education, diagnosis seems to work as a protective factor for both the psychological and school well-being of the student. In fact, a diagnosis offers the opportunity to access specific resources, specific supports, which contrast the risk of psychological distress created by disadvantageous and stressful school experiences (e.g., relational and emotional dimensions and school performance) [67]. In other words, the recognition of the diagnosis and related difficulties, in the Italian context, activates in teachers educational strategies that are closer to the needs of the students and that capitalize on their individual strengths. The students without any clinical diagnosis but with learning difficulties should be considered as having a SLD in order to gain the advantage of these protective regulations, which should allow them to engage in learning strategies that will have an effect on their academic achievement.

Overall, this study shows that when struggling students have the opportunity to be addressed with more personalized teaching methods, such as the SLD group in the Italian context, their well-being experience, their engagement in school activities, and their representations of school climate are as positive as those of their schoolmates. On the contrary, when struggling students do not meet an educational context that supports them, such as the LD group in this study, negative effects on well-being, engagement, and school-climate representation might be observed.

It is worth nothing that reasons for dropping out of school before completion are related to environmental impact and academic achievement, as both categories affect the ability of students with learning difficulties to complete secondary school [68]. Experiencing numerous failures at school can leave students frustrated, generating negative expectations about their performance and a negative impact on their subsequent efforts. Low achievement at school affects the self-image of students with learning difficulties, when such difficulties do not meet an inclusive education and when struggling students are expected to compare themselves (in terms of personal, familiar, and scholastic expectations) with their peers without learning difficulties. These beliefs can intensify the feelings of defeat and frustration already detected in several studies conducted with students with specific learning disorders [69,70]. However, the results of this work suggest that the implementation of an inclusive education, according to the International Classification of Functioning perspective, might reduce such negative effects on well-being experience, engagement in school and school climate perception.

## 5. Strengths and Limitations

Although the results of our study give some clues regarding the condition of struggling students in secondary school, given the relatively small size of each group, the study can be considered only exploratory. A larger sample size would have allowed us to assess relationships within the different variables through more complex statistical techniques, usually applied to verify mediation and moderation effects. However, to the best of our knowledge, this is the first study which compared two different groups of struggling students (with and without diagnosis of specific learning disorders), matched with each other and with a typically developing control group, starting from a screening on a larger study. This is even more significant when considering it was performed in an age range that is typically overlooked for this topic. Therefore, we hope that the present study might be considered a useful ‘stone in the pond’.

## Figures and Tables

**Table 1:** Means and standard deviations for scores at the Raven’s Progressive Matrices and z-scores on the literacy skill tests, for the three groups.

	LD				SLD				Control			
	*M*	*SD*	*MIN*	*MAX*	*M*	*SD*	*MIN*	*MAX*	*M*	*SD*	*MIN*	*MAX*
Raven’s Progressive Matrices	43.57	5.37	37	55	41.85	6.27	39	52	42.9	3.94	37	48
Reading Speed in pseudo-words	−1.05	0.87	−2.3	0.45	−0.98	0.91	−2.89	0.18	0.06	0.71	−1.2	1.2
Reading Accuracy pseudo-words	−0.93	1.65	−4.64	0.7	−0.69	0.92	−2.49	0.51	0.01	0.93	−0.21	1.37
Reading Comprehension	−0.34	0.81	−1.15	1	−0.06	0.95	−1.92	1.3	0.6	0.82	−1	1.3
Accuracy in spelling	−0.12	1.31	−2.94	1.4	−0.34	1.23	−2.65	1.4	0.52	0.65	−0.7	1.2

**Table 2:** Correlations between school climate, well-being and student engagement.

Groups		School Climate	Well-Being	Affective Eng.	Behavioral Eng.
*Control*	Well-Being	0.389			
	Affective Eng.	0.516	0.406	-	
	Behavioral Eng.	0.519	0.165	0.757 **	-
	Cognitive Eng.	0.205	0.321	0.615 *	0.642 *
*SLD*	Well-Being	0.293			
	Affective Eng.	0.313	0.680 **	-	
	Behavioral Eng.	0.503	0.693 **	0.881 **	-
	Cognitive Eng.	0.345	0.423	0.780 **	0.753 **
*LD*	Well-Being	0.792 **			
	Affective Eng.	0.544 *	0.611 *	-	
	Behavioral Eng.	0.546 *	0.694 **	0.752 **	-
	Cognitive Eng.	0.188	0.578 *	0.582 *	0.596 *

* *p* < 0.05; ** *p* < 0.01 (two tales).

**Table 3:** Descriptive statistics and differences between groups (Kruskal–Wallis test) on well-being, student engagement, and school climate.

		LD (*n* = 14)			SLD (*n* = 14)			Control (*n* = 14)			Kruskal–Wallis Test	
	Scales	*M*	*SD*	*Mdn*	*M*	*SD*	*Mdn*	*M*	*SD*	*Mdn*	χ^2^	*p*
Well-Being (CIT)	Relationships	3.35	0.55	3.42	3.56	0.44	3.57	3.27	0.42	3.19	2.74	0.13
	Engagement	3.17	0.57	3.17	3.74	0.44	3.83	3.60	0.75	3.67	6.56	0.02
	Mastery	3.13	0.51	3.30	3.57	0.41	3.70	3.29	0.60	3.47	4.94	0.04
	Optimism	3.74	1.01	4.00	3.74	0.62	3.67	3.45	0.72	3.33	1.95	0.19
	Subjective WB	3.54	0.42	3.56	3.47	0.40	3.44	3.21	0.60	3.28	2.78	0.12
	Autonomy	3.21	0.55	3.00	3.69	0.56	3.67	3.48	0.70	3.33	4.65	0.05
	Meaning	3.24	0.79	3.33	3.48	0.76	3.50	3.14	0.85	3.17	1.18	0.28
Student Engagement (SE)	Affective Eng.	4.31	0.87	4.44	4.61	0.98	4.50	4.85	0.87	4.73	1.18	0.28
	Behavioural Eng.	4.06	0.59	4.00	4.38	0.89	4.29	4.77	0.67	4.68	6.23	0.02
	Cognitive Eng.	4.14	0.88	4.58	4.31	1.34	4.17	4.55	1.08	4.54	1.08	0.29
School Climate (GSCS)	School Connectedness	2.31	0.46	2.50	2.61	0.56	2.50	2.88	0.56	2.70	6.51	0.02
	Peer Social Support	2.77	0.54	3.00	3.04	0.72	3.25	3.22	0.42	3.30	5.24	0.04
	Adult Social Support	2.77	0.58	2.90	2.39	0.88	2.75	2.41	0.55	2.40	2.69	0.13
	Cultural Acceptance	2.23	0.70	2.35	2.46	0.69	2.50	2.11	0.59	2.00	2.34	0.16
	Social/Civic Learning	3.08	0.43	3.00	3.43	0.62	3.50	3.38	0.40	3.40	3.56	0.08
	Physical Environment	2.34	0.84	2.50	1.89	0.76	2.00	1.97	0.74	1.75	2.59	0.14
	School Safety	1.85	0.23	2.00	2.04	0.41	2.00	2.11	0.40	2.00	4.08	0.07
	Peer Victimization	3.16	0.99	3.60	3.61	0.81	4.00	3.31	1.18	4.00	2.11	0.17
	Order and Discipline	2.58	0.51	2.80	2.36	0.72	2.50	2.76	0.54	2.65	1.37	0.25
	Parents’ Involvement	3.24	0.64	3.50	3.64	0.36	3.50	3.11	0.45	3.00	9.24	0.01

## Data Availability

The data presented in this study are available on request from the corresponding author. The data are not publicly available due to the privacy restrictions.
